# Cardiac Calcifications: Phenotypes, Mechanisms, Clinical and Prognostic Implications

**DOI:** 10.3390/biology11030414

**Published:** 2022-03-09

**Authors:** Francesco Vieceli Dalla Sega, Francesca Fortini, Paolo Severi, Paola Rizzo, Iija Gardi, Paolo Cimaglia, Claudio Rapezzi, Luigi Tavazzi, Roberto Ferrari

**Affiliations:** 1 Cardiovascular Department, GVM Care and Research, Maria Cecilia Hospital, Via Corriera 1, 48033 Cotignola, Italy; fvieceli@gvmnet.it (F.V.D.S.); ffortini@gvmnet.it (F.F.); rzzpla@unife.it (P.R.); igardi@gvmnet.it (I.G.); pcimaglia@gvmnet.it (P.C.); claudio.rapezzi@unife.it (C.R.); ltavazzi@gvmnet.it (L.T.); 2 Laboratory for Technologies of Advanced Therapies (LTTA), Department of Translational Medicine, University of Ferrara, Via Fossato di Mortara 64/B, 44121 Ferrara, Italy; svrpla@unife.it; 3 Prevention Centre, University of Ferrara, Corso Ercole I° D’Este 32, 44121 Ferrara, Italy

**Keywords:** hyperphosphatemic calcification, vascular calcification, valvular calcification, inflammatory calcification, extracellular vesicles, Notch

## Abstract

**Simple Summary:**

The clinical relevance of vascular calcifications has increased in recent years, given the aging of the population and increased exposure to risk factors. Tissue calcification is often a point of no return that leaves no room for any medical therapy and limits the possibility of surgical and interventional treatments—a real insurmountable barrier. The diffusion of cardiac imaging methods has made the recognition of cardiac calcifications, at various levels and of variable extent, more and more frequent. The pathogenesis of calcifications is not unique but includes different mechanisms, depending on the specific site and disease, which, in turn, results in different phenotypes. Unfortunately, however, clinicians are not always aware of these different mechanisms and phenotypes. This concise, but in-depth, review explores the different molecular processes and their links with the specific clinical condition, and current therapeutic approaches to counteract calcifications.

**Abstract:**

There is a growing interest in arterial and heart valve calcifications, as these contribute to cardiovascular outcome, and are leading predictors of cardiovascular and kidney diseases. Cardiovascular calcifications are often considered as one disease, but, in effect, they represent multifaced disorders, occurring in different milieus and biological phenotypes, following different pathways. Herein, we explore each different molecular process, its relative link with the specific clinical condition, and the current therapeutic approaches to counteract calcifications. Thus, first, we explore the peculiarities between vascular and valvular calcium deposition, as this occurs in different tissues, responds differently to shear stress, has specific etiology and time courses to calcification. Then, we differentiate the mechanisms and pathways leading to hyperphosphatemic calcification, typical of the media layer of the vessel and mainly related to chronic kidney diseases, to those of inflammation, typical of the intima vascular calcification, which predominantly occur in atherosclerotic vascular diseases. Finally, we examine calcifications secondary to rheumatic valve disease or other bacterial lesions and those occurring in autoimmune diseases. The underlying clinical conditions of each of the biological calcification phenotypes and the specific opportunities of therapeutic intervention are also considered and discussed.

## 1. Introduction

Cardiovascular (CV) calcification is a growing research topic in cardiology, mainly because the deposition of calcium salts is associated with major CV diseases, such as atherosclerosis, valvular diseases, and several hypertrophic cardiomyopathies. Recently, coronary artery calcium measurements have been proposed as the most accurate means for screening or risk assessment for atherosclerotic CV disease [1].

**Figure 1:**
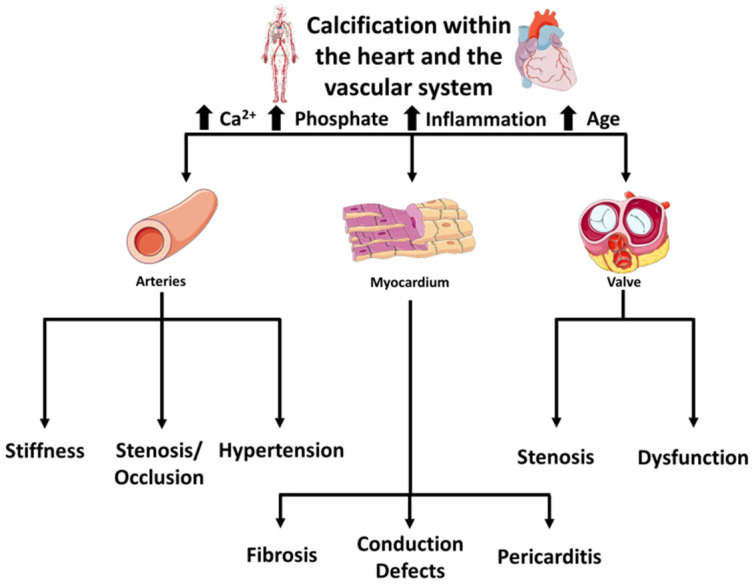
Pathological calcifications and clinical consequences that can occur within the heart and the vascular system.

Calcifications can involve arteries and heart valves, causing stiffness and dysfunction and, less frequently, the myocardium, causing fibrosis, conduction defects, and pericarditis. Figure 1 schematizes the most common pathological calcifications within the heart and the vascular system, the two most relevant being vascular and valvular calcifications. Although sharing risk factors and similar molecular pathways, vascular and valvular calcifications occur in different tissues, react to different stimuli, and represent two distinct biological phenotypes [2].

Differences also exist within the vascular calcifications related to whether calcium deposition occurs mainly in the intimal or medial layer of the arterial wall.

This article summarizes the intracellular processes and pathways controlling hyperphosphatemic calcification, occurring mainly in the media layer of the vessels, and the intima vascular calcification, which is predominantly linked to inflammation. The specific mechanisms of both processes are analyzed in the two most relevant districts, namely, in the arterial wall and the aortic valve. The molecular mechanisms leading to rarer calcification, in response to infections and autoimmune diseases, are also examined. Finally, we consider the different pathophysiological phenotypes in light of the clinical conditions associated with each phenotype, the eventual predictive value, and possible therapeutical implications.

## 2. Diversity in Vascular Versus Valvular Calcification

Although these calcifications share several routes and risk factors, not all patients with calcific aortic stenosis (CAS) present significant coronary artery diseases (CAD), which occur in only 25–40% of CAS cases. This suggests different pathways for the two types of calcifications, which diverge in several aspects.

Firstly, the sites of calcification are different. The valve is a complex tri-layered structure, consisting of collagen, elastin, and proteoglycans, instead of the vasculature’s simple collagen and elastin-rich layer. The valvular leaflets are arranged in three layers: fibrosa, spongiosa, and ventricularis. The leaflets of both the aortic and ventricular surfaces are covered by endothelium. Each of these layers has a distinct structure and function. The fibrosa, made of connective tissue, provides the strength of the leaflet. The spongiosa, made of mucopolysaccharides, facilitates the movement of the valve. The ventricularis, made of elastin, contributes to its flexibility, allowing for changes in shape during opening and closing. Each layer has a sub-population of resident interstitial cells, essential for maintaining homeostasis within the leaflets and avoiding inflammation [3]. The vessels’ walls are constituted mainly by smooth muscles and elastin-rich layers, with only one side covered by endothelium. The resident cells are also different: vascular smooth muscle cells (VSMCs) for the vessels and valve interstitial cells (VICs), essentially fibroblasts, for the valve. These are the cells that, under pathologic conditions, may undergo myofibrogenic [4], osteogenic, and even chondrogenic differentiation [5].

Secondly, the reaction to shear stress is different. The valves are exposed to pulsatile shear stress on the ventricular side and to low and reciprocating flow on the aortic side, as opposed to the arteries’ laminar flow. With the progression of valvular stenosis, shear stress increases dramatically on both sides and activates latent factors that can induce fibrosis and calcification [3]. Of note, bicuspid aortic valves are exposed to greater shear stress than the tri-leaflet valves and, consequently, calcify earlier [6]. Vascular shear stress also drastically increases at the level of intravascular plaque, where calcifications often occur.

Thirdly, the etiology is different. Increased longevity and lower prevalence of rheumatic heart diseases have caused, at least in the industrialized countries, a pattern shift in the valvular calcification: from rheumatic to a degenerative pathology. On the contrary, inflammation and genetic predisposition seem to be prevalent in vascular calcification occurring in CAD and PAD [7,8].

Fourthly, the time courses of valvular versus vascular calcification are different. The time required for valve calcifications is longer than that needed for vascular mineralization, suggesting that the valves are more resistant to calcific insults than the vessels. Table 1 summarizes these differences, which will be described in detail in the following sections.

## 3. Diversity in Medial Versus Intimal Vascular Calcifications

Medial and intimal vascular calcification are defined by etiology, location within the arterial wall, regional distribution, and clinical consequences. Unfortunately, non-invasive imaging is unable to discern the two calcifications; therefore, the knowledge and clinical prevalence of the two types is limited.

**Figure 2:**
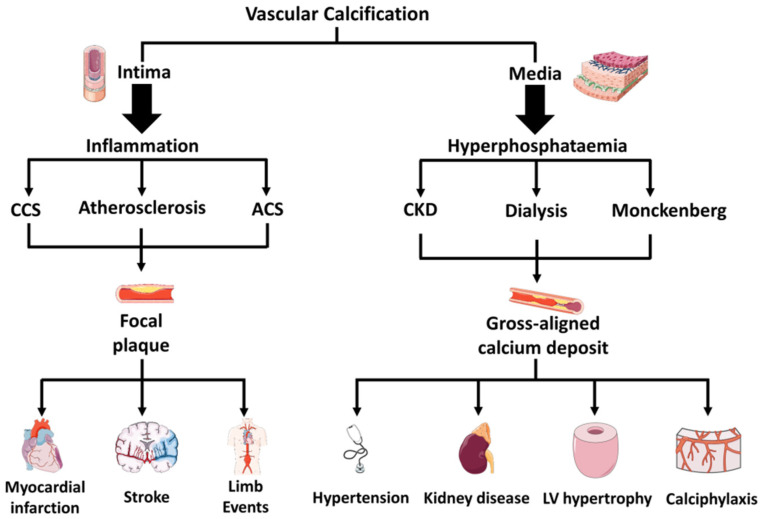
Main differences in calcification occurring in the intimal or media layer of the vessels. CCS = Chronic Coronary Syndrome. ACS = Acute Coronary Syndrome. CKD = Chronic Kidney Disease.

Intimal calcifications are associated with atherosclerosis, which, in turn, is characterized by lipid accumulation, inflammation, fibrosis, and the development of focal plaques. Medial calcifications may also be associated with arteriosclerosis, but to a lesser extent. Commonly, they arise from serum hyperphosphatemia and are specific to chronic kidney disease (CKD). Medial calcifications are associated with the severity of CKD, dialysis vintage, and Monckeberg’s syndrome, and are frequent in diabetes mellitus and advanced age, resulting in increased arterial stiffness that causes hypertension, increased cardiac work, and, eventually, left ventricular hypertrophy [7]. A complication related to CKD and hemodialysis is calcific uremic arteriolopathy, also called calciphylaxis. In this disease, calcium rapidly accumulates in the small arterioles of the adipose and skin tissues, causing clots and ulcers, which can lead to life-threatening infections [9]. Intimal calcifications are associated with CAD or PAD but can also occur in larger arteries, such as the aorta, causing inflammation-driven vascular remodeling [2,10]. Calcium deposits in atherosclerotic plaque are predictive of adverse events, such as myocardial infarction, stroke, or limb events. In particular, micro-calcifications within the fibrous caps increase local stress and the risk of plaque rupture, with consequent development of an acute coronary syndrome [11]. Figure 2 outlines these differences.

## 4. Mechanism of Hyperphosphatemic Medial Calcification

Hyperphosphatemia, associated with CKD, leads to a rapid wall and aortic valve calcification. The histology of arteries from these patients shows gross, aligned mineral deposits, diffused in topographic areas of the vascular wall, typically devoid of lipid accumulation or atherosclerotic lesions (Figure 2).

Two processes essentially achieved maintenance of the serum phosphate within the physiological levels: (i) the regulation of bone formation and absorption; (ii) the control of phosphate excretion in the proximal tubules of the kidney. These processes are finely regulated by parathyroid hormone (PTH), fibroblast growth factor (FGF)-23, and 1,25-dihydroxy-vitamin D (calcitriol), through complex interactions, as discussed later and described in more detail elsewhere [12,13].

It is plausible that the increase in serum phosphate concentration contributes to the direct precipitation of calcium phosphate. This, however, does not happen in every patient with hyperphosphatemia, as numerous physiological mechanisms prevent the growth of calcium phosphate crystals. Therefore, there must be other explanations for calcium deposition. Table 2, at the end of this section, summarizes the agents that favor or contrast the increase in vascular calcification. 

**Figure 3:**
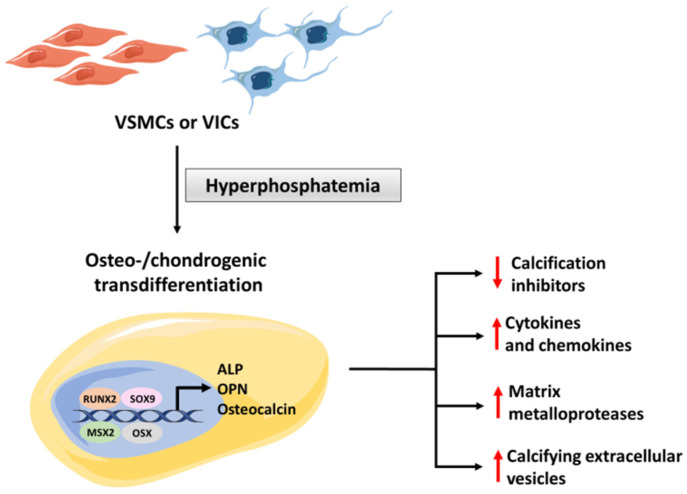
Mechanism of transdifferentiation of vascular smooth muscle cells (VSMCs) or valvular interstitial cells (VICs) into osteoblasts. RUNX2 = Runt-related transcription factor 2; MSX2 = Msh homeobox; OSX = Osterix; OPN = osteopontin; ALP = alkaline phosphatase.

In vitro studies suggest that increased phosphate induces dose and time-dependent, closely regulated changes in vascular and/or valvular cells, discussed below and schematized in Figure 3.

### 4.1. The Role of Osteogenic Transdifferentation

The presence of a pro-calcific milieu mediates the transdifferentiation of VSMCs (in the vessels) or VICs (in the valve) into the osteochondrogenic cells. Studies in transgenic mice have shown that the Nuclear Factor-kappa B (NF-κB) pathway plays an essential role in the transdifferentiation of VSMCs and VICs [14,15]. This process is mediated by osteogenic transcription factors, such as Runt-related transcription factor 2 (RUNX2), Msh homeobox (MSX2) and Osterix (OSX), and chondrogenic (SOX9) transcription factors [5], which drive the expression of osteochondrogenic-specific proteins, including osteopontin (OPN), osteocalcin, and alkaline phosphatase (ALP) [16]. Mutations in the NT5E gene, which encodes for CD73 protein, cause lower-extremity medial artery calcifications, which resemble those of PAD [17]. Studies in vitro and in animal models have shown that CD73 deficiency promotes stem cell differentiation into the osteogenic lineage and that defective CD73-mediated signaling causes an increase in TNAP activity, sufficient to induce calcification [8,18]. Once established, osteoblasts act as bone-forming cells, producing a collagen matrix with calcium and phosphate secretion. This process is mediated by several mechanisms, including: (i) decreased production of calcification inhibitors; (ii) release of calcifying extracellular vesicles; (iii) expression of matrix metalloproteases (MMP), with consequent matrix remodeling; (iv) release of inflammatory cytokines and chemokines [5]. Thus, the process is very dynamic and is highly regulated (Figure 3).

### 4.2. Regulatory Role of FGF-23 and Klotho

FGF-23 is a fibroblastic factor, primarily produced in the bone. Its principal function is to directly control blood phosphate and calcium concentrations by promoting renal phosphate excretion, and indirectly, by inhibiting the synthesis of calcitriol [13]. In animal models, genetic ablation or antibodies neutralizing FGF-23 cause hyperphosphatemia and tissue calcification [19]

In CKD patients, FGF-23 levels are increased and associated with mortality and cardiovascular events [7,20]. However, it is still debated whether changes in FGF-23 levels inhibit or promote vascular calcification [7,21]. A crucial unanswered question is whether FGF-23 can act directly on vascular cells to influence calcification. The evidence in this regard is conflicting; some studies have shown that FGF-23 protects SMC from calcification [22,23], others have shown pro-calcifying effects [24], still, others have not shown any effect of FGF-23 on calcification [25,26]. The reasons for these divergent findings are not easy to determine; different experimental settings may have led to inconsistent results or even that there are no direct effects of FGF-23. Future studies are needed to clarify this critical point.

The activity of FGF-23, in turn, depends on Klotho, a co-receptor, essential for the binding of FGF-23 to its receptor, FGFR [27]. Klotho knock-out mice display increased serum phosphate and extensive vascular calcification [28], while adding exogenous Klotho to VSMCs directly suppresses osteogenic transdifferentiation [29]. Also, in human aortic valves, Klotho suppresses high phosphate-induced osteogenic response by inhibiting the osteochondrocytic transcription factor SOX9 [30]. Unlike those on FGF-23, findings regarding Klotho converge, in showing its role in suppressing vascular calcification.

### 4.3. Regulatory Role of MGP, Fetuin-A and Calciprotein Particles

Vitamin K-dependent matrix Gla-(γ-carboxyglutamate) protein (MGP) and fetuin-A also play a regulatory role in vascular calcification. VSMCs secrete MGP in the tunica media, where it acts as a calcification inhibitor. Mutations in the MGP gene cause Keutel syndrome, in which patients develop calcification in soft tissues throughout the body, including vascular vessels [31]. MGP knockout mice develop massive vascular calcification in their first weeks of life and die within two months because of vessel rupture [32]. Fetuin-A is a glycoprotein secreted by the liver. It is present in relatively high concentrations in the serum and can be uptaken by VSMCs [33]. Fetuin-A-deficient mice, exposed to hyperphosphatemia, display widespread vessel calcification [34]. In vitro and in vivo studies have shown that fetuin-A binds and removes calcium and phosphate, which is the basis for its anti-calcifying activity [35]. Fetuin-A and MGP cooperate to store excessive calcium and phosphate into amorphous spherical particles, sized 50 to 500 nm in diameter, called calciprotein particles (CPPs) [36]. These particles, generally referred to as “*primary CPPs*”, facilitate the clearance of calcium and phosphate and, therefore, protect from pathological calcification. However, CPPs have a bivalent effect. When hypercalcemia or hyperphosphatemia persist, CPPs are transformed into calcium hydroxyapatite (*needle-shaped*) particles, called “*secondary CPPs* ”, which promote vascular calcification [37]. More specifically, secondary CPPs can enter vascular endothelial cells and induce apoptosis [38], expression of leukocytes adhesion molecules, endothelial-to-mesenchymal transition (EndMT), thus, impairing the anti-calcific properties of the endothelial monolayer [39]. The result is enhanced osteochondrogenic transdifferentiation and calcification [5].

### 4.4. Regulatory Role of Extracellular Vesicles

Extracellular vesicles (EVs) are a heterogeneous group of secreted vesicles, including matrix vesicles and exosomes, regulating mineral homeostasis. Patidar and colleagues showed uremic serum from patients with CKD-induced calcifications of cultured VSMCs and suggested that pro-calcific EVs are present in their serum [40]. Vascular calcification can be triggered by the secretion of pathological EVs with calcifying properties [41], also termed calcifying micro-vesicles (CMVs). CMVs, secreted by VSMCs, in a high phosphate environment, exhibit a specific proteomic profile [42] and resemble those released by osteoblasts [43]. Once released in the extracellular space, CMVs aggregate by annexin-dependent tethering and bind to matrix collagens to form nucleation sites for calcification [44]. 

**Table 2:** Promoters and inhibitors of vascular calcification. CMVs = calcifying micro-vesicles; CPPs = calciprotein particles; FGF-23 = Fibroblast Growth Factor 23; MGP = Vitamin K-dependent matrix Gla- (γ-carboxyglutamate) protein; NF-kB = Nuclear Factor- kappa B; OPN = Osteopontin; OSX = Osterix; RUNX2 = Runt-related transcription factor 2; SOX9 = ostheochondrocitic transcription factor; TNAP = tissue nonspecific alkaline phosphatase; VIC = Valve interstitial cell; VSMC = Vascular Smooth Muscle Cell.

Role	Actor	Description	Reference
Promoters of vascular calcification	TNAP	TNAP is an ectoenzyme that catalyzes dephosphorylations. Its activity releases free phosphate, which promotes mineralization.	[2]
	RUNX2	RUNX2 is a key transcription factor controlling osteoblast differentiation.	[5]
	OSX	OSX is a transcription factor necessary for osteocyte differentiation and bone formation	[5]
	NF-kB	NF-kB pathway plays an essential role in the transdifferentiation of VSMCs and VICs into osteochondrogenic cells.	[14,15]
	Osteocalcin	Osteocalcin is a hormone and osteogenic marker produced by osteoblast-like cells.	[16]
	SOX9	SOX9 is a transcription factor associated with osteoblast-like transdifferentiation	[30]
	Secondary CPPs	Secondary CPPs are calcium hydroxyapatite nano-particles produced from primary CPPs under persistent hypercalcemia or hyperphosphatemia.	[36]
Inhibitors of vascular calcification	FGF-23	FGF-23 regulates phosphatemia by controlling renal phosphate excretion. Its role in vascular calcification is still debated.	[7]
	Klotho	Klotho is a co-receptor essential for the binding of FGF-23 to its receptor. In addition, Klotho directly suppresses osteogenic transdifferentiation.	[30]
	MGP	MGP is a Gla-containing protein which binds calcium. It is secreted by VSMCs and acts as a potent inhibitor of vascular calcification.	[31]
	Fetuin-A	Fetuin-A is a glycoprotein secreted by the liver which binds calcium and phosphate, collaborating with MGP to prevent calcium precipitation in tissues.	[35]
	Primary CPPs	Primary CPPs are amorphous particles sized 50 to 500 nm that facilitate the clearance of calcium and phosphate, protecting from pathological calcification.	[36]
	CMVs	CMVs can promote or inhibit mineralization, depending on the phenotype of their originating cells and on the extracellular milieu.	[41]

## 5. Mechanism of Inflammatory Intima Vascular Calcification

Intimal calcifications are associated with atherosclerosis and, thus, are characterized by lipid deposition, a robust inflammatory response, and are favored by the same risk factors as for atherosclerosis. It is well known that atherosclerotic plaques can vary widely in composition, including calcium content, and this is known to affect plaque stability and, thus, disease course. This section will focus on the mechanisms that link inflammations to vascular calcifications.

Inflammation-driven intimal calcifications are different from medial calcification. Initially, they appear as spherical or ellipsoidal micro-calcifications, arising from the coalescence of calcifying EVs. Later, they merge to form larger macro-calcifications that may stabilize atherosclerotic plaques but disrupt aortic valve mechanical and functional properties [37].

**Figure 4:**
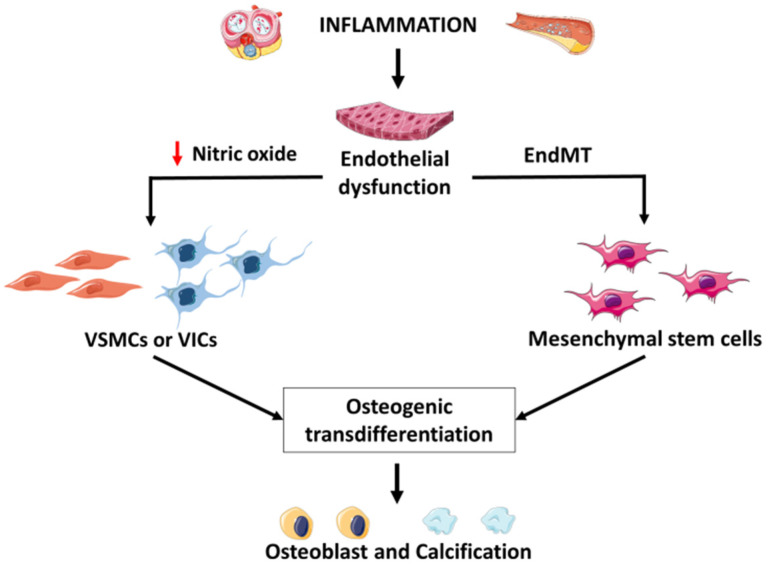
Mechanism of inflammatory vascular and valvular calcification. Vascular smooth muscle cells = VSMCs; valvular interstitial cells = VICs; endothelial-to-mesenchymal transition = EndMT; VSMC = Vascular smooth muscle cell; VICs = valvular interstitial cells; EndTM = endothelial-to-mesenchymal transition.

Studies in vitro and in animals have shown that the inhibition of inflammation prevents intima calcification. At the same time, pro-inflammatory stimuli exacerbate it through mechanisms involving apoptotic cell death and activation of the osteogenic pathways, secondary to an increased expression and activity of tissue non-specific alkaline phosphatase (TNAP) [2]. Nevertheless, intimal calcification is not a simple one-way causal relationship, but the endpoint of a complex inflammation-driven remodeling of the vascular wall, involving interactions among endothelial cells, VSMCs, and immune cells, as schematized in Figure 4 [2].

### 5.1. Role of Endothelial Cells

At the onset of atherosclerosis, plasma lipoproteins accumulate in the sub-endothelial space, triggering inflammatory responses, with the expression of leukocytes’ adhesion molecules and impairment of endothelial function. Under physiological conditions, nitric oxide (NO), produced by endothelial nitric oxide synthase (eNOS), prevents the transdifferentiation of VSMCs into osteoblastic cells by inhibiting transforming growth factor-β (TGF-β) signaling through a cGMP-dependent pathway [45]. On the contrary, dysregulation of eNOS induces oxidative stress, with the production of oxidized low-density lipoproteins (LDL) and phospholipids, which, in turn, drive VSMCs apoptosis and the consequent release of apoptotic bodies, which act as a scaffold for further calcification [46]. Furthermore, in aortic valves, the lack of endothelial NO triggers NF-κB and induces Dipeptidyl peptidase 4 (DPP-4) expression in VICs, which, in turn, determines osteogenic differentiation [47].

In response to pro-atherogenic stimulus, endothelial cells can engage in a de-differentiation program, called endothelial-to-mesenchymal transition (EndMT). Endothelial cells lose their characteristics during this process as they begin to express multipotent stem cell markers. In the context of atherosclerosis, it is well documented that vascular calcification is accompanied by EndMT [48]. Significantly, in animal models of vascular calcification, the expression of markers of multipotency, such as SOX2, NANOG, OCT4 (octamer-binding transcription factor 4), precedes osteogenic transition [48]; conversely, endothelial-specific deletion of those factors contrasts calcification [49]. This indicates that endothelial cells can differentiate into osteogenic cells, transitioning through a mesenchymal phenotype.

Several studies have also implicated EndMT in the pathology of CAVD. In aortic valves, disturbed shear stress triggers the expression of UBE2C (ubiquitin E2 ligase C), which, in turn, triggers EndMT, promoting aortic valve calcification [50]. The involvement of shear stress and development-related pathways in EndMT-mediated calcification is well known; we will focus on this in the next section.

### 5.2. Regulatory Role of Shear Stress and of Notch and Wnt Signaling

The Notch and Wnt pathways are necessary for cell-to-cell communications and mediate stimuli derived from shear stress [51]. Physiological, laminar shear stress activates Notch1 in endothelial cells, inhibiting the expression of inflammatory and pro-calcific genes [52,53] and inducing the expression of anti-calcific MGP [54]. Dysregulation of shear stress, with a reduction in Notch1 activity in areas subjected to disturbed flow, such as the aortic arch and coronary bifurcations, promotes atherosclerosis and calcification [55,56]. However, the link between mutations of Notch1 and aortic valve stenosis is different [56,57,58]. In endothelial cells and VICs, isolated from patients with bicuspid and tricuspid aortic valves [59], the activation of Notch1 signaling no longer has anti-calcification properties, but it enhances bone morphogenetic protein 2 (BMP2) responsiveness of the MSX2 gene, to induce osteogenic VSMC differentiation and calcification [60]. Thus, more studies are needed to elucidate the opposite effects of the Notch pathway in vascular and valvular calcification.

Impairment of Wnt signaling is involved in vascular calcification [55,61] but with effects strictly dependent on the cell type. In endothelial cells, active Wnt signaling prevents EndMT, thus, converting the endothelium into a state leading to vascular and valvular calcification [62]. In mesenchymal cells, Wnt promotes myofibroblast [63] and osteogenic differentiation [64], interacting with bone morphogenetic proteins (BMPs) signaling [65] and with the transcription factor MSX2 [66].

### 5.3. Regulatory Role of Immune Cells

The innate immune system is the first defense activated by pathogens, which are recognized by toll-like receptors (TLRs), expressed on the membrane of innate immune cells. These receptors, in particular TLR2 and TLR4 isoforms, are implicated in calcification, as their activation amplify the inflammatory responses [67].

In atherosclerotic plaque, inflammatory response drives further macrophage polarization towards a pro-inflammatory phenotype; these macrophages are termed M1. In valves and vessels, M1 macrophages release pro-inflammatory cytokines, including tumor necrosis factor-α (TNF-α), interleukin (IL)-1β, IL-6, and IL-18, which increase the expression of osteochondrogenic factors and subsequent VSMCs osteoblastic transdifferentiation [68,69]. In addition, M1 macrophages also secrete proteinases, such as MMP-2, MMP-9, or cathepsins that lead to the degradation of extracellular matrix proteins and are implicated in intimal, medial, and aortic valve calcification [70,71]. The early innate immune response then triggers a late adaptive response, also involved in calcification. T helper (Th) lymphocytes are the primary cells of late adaptive immunity. Th 1 lymphocytes are commonly present in plaques, where they secrete inflammatory cytokines that promote the progression of atherosclerosis but are not involved in cardiovascular calcification. Conversely, Th2 cells stabilize atherosclerotic plaques, produce IL-4, and promote VSMCs osteoblastic transdifferentiation [72]. Other cells of adaptive immunity, such as B-cells, are present in the area of calcification, but their role is unknown.

### 5.4. Regulatory Role of Clonal Hematopoiesis of Indeterminate Potential

Clonal hematopoiesis of indeterminate potential (CHIP) is defined by the presence of clones, carrying a mutation associated with a blood neoplasm, but without evident hematological disorders [73]. Studies in animal models showed that mutations associated with CHIP activate immune cells, promoting inflammation and coronary calcification [74]. Human CHIP carriers have an increased risk of CAD and, possibly, of valvular diseases, such as calcific aortic stenosis [75]. In support of this hypothesis, patients with aortic stenosis have a higher CHIP prevalence than age-matched patients with or without CAD [76]. In addition, CHIP carriers show an increased risk of death than non-carriers after a successful valve replacement [76]. However, a causal relationship between CHIP and valve calcification has not been established yet.

## 6. Medial Calcification in Diabetes

In this regard, the diabetic patient represents a challenging mix of clinical and pathogenetic problems. In adults with type II diabetes, affected by CAD, the probability that peripheral arterial disease coexists is in the range of 20%. Conversely, in the presence of symptomatic peripheral arterial disease, the probability of CAD is 30%, which rises to 40–50% in the case of carotid pathology. Allison and colleagues found a difference in the prevalence of calcification among different vascular beds, in 4544 patients who had undergone whole-body CT scans during an 8-year follow-up. Among them, calcification in the coronary artery had the highest prevalence (55.8%), followed by the abdominal aorta (54.8%), while calcification in the carotid artery had the lowest prevalence rate (32.2%) [77]. In a multiethnic study of atherosclerosis (MESA), the association between abdominal aortic calcification (AAC) and diabetes was the strongest among all vascular beds [78]. Patients with calcification in the carotid, coronary, and iliac arteries have a significantly higher body mass index. In addition, patients with calcification in any vascular bed other than the carotid artery have a higher probability of a family history of cardiovascular disease [79].

In type II diabetes, the distribution of calcifications in the arterial wall and their pathogenetic mechanisms have profound similarities with CKD. Recent evidence suggests that medial calcification in diabetes is an active, cell-mediated process, similar to that observed in patients with end-stage renal disease (ESRD). VSMCs’ several bone matrix proteins act to either facilitate or regulate the calcification process. Therefore, the phenotypic and the molecular fingerprints of medial calcification in patients with diabetes and patients with chronic kidney disease, including those on dialysis, are strikingly similar. While disturbances in divalent ion homeostasis have been proposed to play a role in the calcification of the media in patients with chronic kidney disease, patients with diabetes have an apparently intact bone and mineral metabolism.

CKD, as well as diabetes, are now recognized as pro-inflammatory states [80,81]. Several studies have shown that the Advanced Glycation End-Products (AGEs)/Receptor for AGEs (RAGE) signaling pathways play a crucial role in the hyperglycemia-mediated vascular calcification switch of VSMCs to osteoblast-like cells [82,83]. This transition is mediated by p38 mitogen-activated protein kinase (MAPK) [84]. Under hyperglycemic conditions, AGEs increase the Serine/threonine-protein kinase (SGK1) expression in VSMCs, leading to the osteogenic transdifferentiation and calcification of VSMCs [85]. Hyperglycemia-induced AKT (Protein kinase B) post-translational modification promotes the activation of this pathway, which, in turn, leads to increased Runx2 transcription and, consequently, vascular calcification [86]. In diabetes, VSMCs calcification is associated with cellular senescence, oxidative stress, DNA damage and is characterized by decreased expression of sirtuin 1 (SIRT1) [87,88]. Recently, it has been shown that, in vitro, SIRT1 activity reduces these processes and attenuates VSMCs calcification, indicating a potential therapeutic role of this pathway [88].

## 7. Drug-Induced Vascular Calcification

In recent years, some pharmacological treatments have emerged to promote the calcification of vessels and valves as a side effect.

### 7.1. Warfarin

Several studies have shown that anticoagulant therapy warfarin is associated with increased calcification in various districts, including coronary arteries [89], carotids [90], lower-extremity arteries [91], abdominal aorta, and heart valves [92]. Warfarin is a competitor of vitamin K, and there is evidence that it accelerates calcification by affecting vitamin K-dependent MGP activation, hence, reducing the physiological anti-calcific properties of this protein [93]. Moreover, it has been recently shown that warfarin can interact with SOX5 and SOX9, promoting osteogenic markers’ expression in VICs [94].

### 7.2. Statins

Lipid-lowering therapy with statins effectively attenuates the progression of atherosclerosis and reduces cardiovascular mortality. Nevertheless, the impact of statins on vascular calcification is still debated as, paradoxically, some studies have shown an increase in vascular calcification in patients taking statins [95]. Importantly, this increase in calcification is not accompanied by increased mortality [96]. Statins promote coronary artery calcification, regardless of their regressive effects on the plaque, suggesting that they stabilize plaque beyond their effects on lipids. [96]. This hypothesis seems to be confirmed by a recent study in dyslipidemic mice, which showed that pravastatin treatment affects the microarchitecture of the calcium deposits and, thus, stabilizes the plaque [97].

### 7.3. COX-2 Inhibitors

We, and others, have found that celecoxib, a COX-2 inhibitor and nonsteroidal anti-inflammatory drug used to treat musculoskeletal pain and arthritis, induces VICs trans-differentiation into myofibroblasts [4] and is associated with an increased risk of CAVD [98].

## 8. Calcification Caused by Infections or Autoimmune Disorders

### 8.1. Infections

Rheumatic heart valve disease (RHVD) is caused by an abnormal immune response to Streptococcus infection. Although RHVD is rare in developed countries, it is the leading cause of CV death in children and young adults in low and middle-income nations. RHVD is characterized by high levels of circulating inflammatory mediators, such as IL-6 and TNF-α, that are strongly correlated with the severity of the valvular malfunction and calcification [99]. Furthermore, the few available studies support the concept that rheumatic calcification is not just a passive process, but consists of a series of molecular events, including the differentiation of VICs into osteoblasts and neo-angiogenesis [100].

Other bacterial infections, such as periodontitis, a risk factor for CAD, have been linked to vascular calcification [101,102]. Equally, *Helicobacter pylori*, the causative agent of various gastrointestinal disorders, is associated with serum antibodies against heat shock protein 65, which correlates with the degree of coronary calcification [103,104].

### 8.2. Autoimmune Diseases

The association between autoimmune diseases, chronic inflammation, atherosclerosis, and vascular calcification has been known for a long time [105]. Rheumatoid arthritis (RA) is characterized by systemic inflammation and early-onset diffuse calcification of different vascular beds, predictors of CV morbidity and mortality [106]. In RA patients, high serum levels of IL-6 and TNF-α and neopterin, a marker of monocytes and macrophages activation and autoantibodies against citrullinated protein, are all independently associated with coronary calcification [107,108].

Systemic lupus erythematosus (SLE) is another autoimmune disease that causes widespread inflammation and calcification of the coronary arteries, aorta, and heart valves [109,110]. In SLE patients, vascular calcification is associated with elevated levels of anticardiolipin and aβ2GPI antibodies of the IgG class [111], while calcification of the aortic valve is associated with antiphospholipid antibodies [112]. Thus, in the context of autoimmune disorders, such as RA and SLE, chronic inflammation and autoantibodies against vascular and valve antigens play a role in the calcification process.

## 9. Therapeutic Approaches

Despite consistent research for drugs targeting several molecular intermediates of calcification, the results, so far, are disappointing. However, new possible therapeutic opportunities for both medial and intimal calcification are under investigation.

### 9.1. Treatment of Hyperphosphatemic Medial Vascular Calcification

(a) Vitamin K: Vitamin K is required to carboxylate MGP and activates its anti-calcification activity. A trial (VitaVasK) is testing the hypothesis that supplementation of vitamin K1 counteracts coronary and aorta calcification. The results are expected soon (ClinicalTrials.gov Identifier: NCT01742273, accessed on 20 January 2022).

(b) Magnesium: Animal studies and small human trials showed that magnesium might inhibit the development of phosphate-induced calcifications, in uremic rats or patients with advanced CKD. An open-label randomized control trial, on just less than 100 patients with CKD stages 3 and 4, was stopped early, as the progression of coronary artery calcification (CAC) was substantially reduced in the treated arm with magnesium oxide [113]. The data are promising but given the open-label design and the small number of participants, they need to be confirmed.

(c) Phosphate binders: Phosphate binders can contain calcium (acetate, carbonate) or not (sevelamer, lanthanum, magnesium). In patients subject to dialysis, the use of calcium-containing binders is associated with higher rates of vascular calcification [114]. The use of calcium-free phosphate binders in these patients is controversial [7]. The same uncertainties hold for the non-dialysis CKD population. Two trials have been set up: the COMBINE, testing the combination of lanthanum and nicotinamide, which was inconclusive [115], and the IMPROVE-CKD, comparing lanthanum to placebo in 488 patients, followed for 96 weeks, which is still ongoing.

(d) SNF472-MYO-Inositol Hexaphosphate: SNF472, also known as IP6, is a hexasodium salt of the active ingredient myo-inositol hexaphosphate or phytate. Pre-clinical studies in rodents yielded promising results [116]. So did CALYPSO, a phase II trial in 274 dialytic patients, randomized to SNF472 (in two doses) or placebo. At 12 months, treated groups showed a significant slowing in the progression of CAC [117]. More extensive studies are necessary to establish safety and efficacy.

(e) Denosumab: Denosumab is a human IgG2 monoclonal antibody used in osteoporosis patients to block bone reabsorption of calcium and phosphate, by inhibiting the link of RANKL with the membrane of osteoblasts and preventing the activation of osteoclasts. It has reduced calcium deposition in the aorta with a poorly understood mechanism and, in vitro, is a potential inhibitor of VICs calcification [3]. Recently, a small observational study showed that denosumab reduces calcification of the aortic arch in CKD patients undergoing hemodialysis [118]. In contrast, in aortic stenosis patients, denosumab is not effective in reducing the progression of aortic valve calcification. [119].

(f) Sodium thiosulphate: Sodium thiosulfate is an antioxidant and chelating agent, used to prevent cisplatin toxicity and treat cyanide poisoning. Sodium thiosulfates have been used to treat calciphylaxis with promising results [120]. In addition, in a small randomized controlled trial, sodium thiosulfate reduced calcification in the iliac arteries and heart and reduced arterial stiffness and carotid intima-media thickness [121].

(g) Vitamin D and calcimimetics: Vitamin D plays a pivotal role in calcium metabolism. Experimental and clinical studies have revealed an association between vitamin D deficiency and cardiovascular diseases (CVD) [66], and that low doses of calcitriol and paricalcitol may reduce vascular calcification [74]. Calcimimetics increase parathyroid cells’ sensitivity to calcium, inhibiting PTH release and reducing circulating calcium. In a randomized clinical trial, low-dose vitamin D, combined with cinacalcet, a calcimimetic, decreased coronary and aortic valve calcification in hemodialysis patients [122].

(h) Bisphosphonates: Bisphosphonates are used to treat osteoporosis in clinical practice, as they effectively prevent bone loss. In vitro and in animal models of CKD, bisphosphonates are protected against vascular calcification, raising interest in their possible use in vascular calcification prevention [123]. However, clinical trials have shown conflicting results, and whether bisphosphonates may have a role in preventing vascular calcification is still debated [124].

(i) TNAP and NF-kB inhibitors: As previously described, TNAP is implicated in several processes leading to vascular calcification. Pre-clinical studies have provided proof-of-concept that TNAP inhibition may be used to contrast calcification [125,126]. However, since TNAP has several essential functions, in addition to its established role in controlling physiological and ectopic mineralization, the effect of TNAP inhibition on these functions is not clear yet. Further studies are needed to evaluate these crucial aspects and, thus, the feasibility of this approach [127]. Similarly, the NF-kB pathway controls the expression of genes involved in the osteogenic transition, as previously described. Some approved drugs have shown inhibitory activity against NF-kB [128], hence, the idea of exploiting these molecules to counteract the vascular calcification is intriguing. However, no clinically approved drug has been specifically developed to inhibit NF-kB yet [129]. Therefore, further studies are needed to evaluate whether the pharmacological inhibition of NF-kB may become a viable option.

### 9.2. Treatment of Inflammatory Intimal Vascular Calcification

The role of lipid-lowering agents, together with angiotensin converting enzyme inhibitors (ACEi), in reducing the progression of atherosclerosis and CVD, beyond cholesterol and blood pressure lowering, is well established [130]. The underlined molecular effects are generally called “pleiotropic” and are related to several possibilities, including a reduction in endothelial apoptosis and increased regeneration, thus, maintaining, intact, the endothelial layer or a favorable genetic action. These mechanisms prevent coronary calcification.

Lipid lowering has also been proposed [131] as a therapeutical possibility to counteract calcific aortic stenosis. In this case, the situation is more complex than in CAD. Mendelian randomization studies have indicated LDL-cholesterol as an important risk factor for aortic stenosis [132,133]. However, three well-conducted randomized control trials could not find any benefit of lowering LDL-cholesterol with statins. There are different explanations for such counterintuitive results, including too short duration of the trials, pro-osteogenic properties of statins, and an increase in lipoprotein (a) [LP(a)] levels. The latter is considered a therapeutical target, as it carries oxidized phospholipids, which promote calcification. The issue, however, is complicated. A post-hoc analysis of the FOURIER trial argues against a role of Lp(a) in aortic stenosis, while the Safe Heart Registry confirms a positive role, at least in familial hypercholesterolemia [134]

An anti-calcification role of Proprotein Convertase Subtilisin/Kexin type 9 (PCSK9) has been confirmed in animal studies, suggesting that a stronger cholesterol reduction with PCSK9 might be needed. Trials with PCSK9 are underway, and we will have to wait before coming to a definitive conclusion.

CANTOS and LoDoCo2 [135,136] studies provided clear evidence that modulating the inflammatory response with canakinumab or colchicine effectively reduces atherosclerotic disease and cardiovascular events. Given the close relationship between inflammation, atherosclerosis, and vascular calcification, it can be hypothesized that immunotherapy may also reduce vascular calcification.

## 10. Conclusions

Vascular, and probably valvular, calcifications are not new. They existed at the time of the Egyptians, as demonstrated by computed tomography of the mummies [137], in line with the hypothesis that age and inflammation, more than nutritional habits, are pathogenic factors.

Through the years, our understanding of calcification has shifted from a simple deposition of minerals to an active, highly regulated cell-mediated process, which represents a common complication in CKD and CVD, diabetes mellitus, valvular disease, and aging. Today, it is clear that CV calcifications are multifaceted disorders, occurring in different milieus, due to different pathologies. Hyperphosphatemia is a determinant of vascular calcification in CKD, while inflammation causes calcification associated with atherosclerosis. These findings marked how different pathological phenotypes exist, not only between vascular and valvular calcifications, but even within the same vessel. Therefore, the first take-home message is that the way forward is difficult but not impossible. The second message is that calcium is involved in several aspects of human life and is pervasive in human fluids. Slight shifts in its homeostasis can result in its mineralization. Unfortunately, the third message is that experimental models mimicking the different human pathological conditions are lacking.

Despite these difficulties, progress in detecting calcification with imaging modalities, as coronary CT with the Agatston score, in the near future, will allow us to reliably and quantitatively distinguish calcification among the different phenotypes.

Several therapeutic agents have been identified but can only partially slow down the progression of vascular calcification. As of today, no therapeutic approach can halt the progression of calcification and, for AS patients, the only available option is the replacement of the valve. Preclinical research shed further light on this multifaceted, unsolved clinical problem and has led to the identification of previously unknown mechanisms promoting calcification that could be targeted. These include: (1) the retinoic acid receptor, which increases MGP while decreasing TNAP [138]; (2) the endoplasmic reticulum stress, which induces vascular calcification by releasing Grp78-loaded EVs [139], and (3) sortilin, which is involved in the traffic of pro-calcific EVs [140]. Repositioning drugs, such as Sitagliptin, an inhibitor of DDP4 used in the treatment of type 2 diabetes mellitus, could be a promising approach to interfere with the progression of CAVD [47]. Ongoing multi-omics studies, conducted in experimental models and patients, along with high-resolution imaging methods and Artificial Intelligence approaches [141], will help to identify a specific molecular signature of calcification, leading to new therapeutic targets.

The Egyptians did not have our multidimensional technologies and knowledge. We do, and we should capitalize on this to find therapeutical opportunities to prevent and halt both vascular and valvular calcifications.

## 11. Limitations

The aim of this Review was to highlight the heterogeneity of cellular and molecular mechanisms, underlying calcification in multiple pathologies and tissues, which hampers our efforts to identify a treatment strategy for this disease. To make the Review of broad interest, while keeping it focused, we chose not to discuss in detail the molecular and cellular mechanisms of calcification associated with each pathology, including differences in local milieu driving calcification at different anatomical sites (for a detailed Review on this last topic, the reader is referred to [16]). For the same reason, we left out a detailed discussion of the steps leading to atherosclerosis, even though strongly intertwined with intimal calcification.

## Figures and Tables

**Table 1:** Main differences between valvular and vascular tissues.

Characteristics of	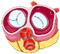 Valves	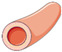 Arteries
STRUCTURE	3 LAYERS: Fibrosa, Spongiosa, Ventricularis	2 LAYERS: Collagen, Elastin
RESIDENT CELLS	FIBROBLAST (VICs)	SMOOTH MUSCLE (VSMCs)
PATHOLOGY	DEGENERATIVE	INFLAMMATORY
TIME COURSE FOR CALCIFICATION	LONG	SHORT

## Data Availability

Not applicable.
